# A Stability Indicating HPLC Method to Determine Actual Content and Stability of Nicotine within Electronic Cigarette Liquids

**DOI:** 10.3390/ijerph15081737

**Published:** 2018-08-13

**Authors:** Mahima Bansal, Manisha Sharma, Chris Bullen, Darren Svirskis

**Affiliations:** 1 School of Pharmacy, Faculty of Medical and Health Sciences, The University of Auckland, Auckland 1023, New Zealand; m.bansal@auckland.ac.nz (M.B.); manisha.sharma@auckland.ac.nz (M.S.); 2 National Institute for Health Innovation, The University of Auckland, Auckland 1010, New Zealand; c.bullen@auckland.ac.nz

**Keywords:** nicotine, HPLC, stability-indicating, e-cigarettes, e-liquids

## Abstract

(1) Background: Despite the growing use of e-cigarettes, in most countries, there is no regulation covering manufacturing standards of the solution (‘e-liquid’), leading to concerns over the accuracy of labelling and stability of the products under a range of conditions. Following the United States (US) Food and Drug Administration (FDA) requirements for manufacture of e-liquids, we aimed to develop a simple high-performance liquid chromatography (HPLC) method to determine nicotine content in nicotine-containing e-liquids, even in the presence of degradation products; (2) Methods: We developed an HPLC method to quantify nicotine in the presence of the two major constituents of all e-liquids, glycerine and propylene glycol, and in the presence of degradation products; (3) Results: Our HPLC method performed strongly and was validated according to international guidelines. For the e-liquids tested, nicotine content levels were all higher than labelled (up to 117.9 ± 1.87% of the labelled content). While nicotine was shown to be unstable at 60 °C, it was stabilized at this temperature in the e-liquid formulations for up to 10 days; and (4) Conclusions: The HPLC method is suitable for adoption by laboratories to determine the actual content and stability of nicotine-containing products. The higher than labelled nicotine levels in e-liquids raises clinical and public health concerns.

## 1. Introduction

Electronic cigarettes (e-cigarettes) were first introduced in China in 2004 as a safe cigarette substitute [1,2]. E-cigarettes comprise a battery and coil to heat and aerosolize a solution (‘e-liquid’) contained in a cartridge or refillable reservoir (‘tank’) for inhalation. Typically, e-liquid is a solution of pure nicotine dissolved in propylene glycol, vegetable glycerine, and flavors [1]. Due to the presence of such simple constituents and lack of combustion in the process of aerosolization, e-cigarettes are considered a safer alternative to tobacco smoking [3]. However, e-liquids are subjected to very high temperatures up to 350 °C during vaporization, and this is sufficiently high to induce chemical reactions between different ingredients and result in the formation of degradation products [4]. A handful of clinical trials have found e-cigarettes to be modestly effective at helping smokers to quit with few reported adverse effects after several months use [5,6]. However, various issues related to labelling and content accuracy have been reported in the literature. Some e-liquids labelled as containing no nicotine were found to have higher concentrations of nicotine [7,8,9]. Meanwhile, Svensson et al. analyzed 20 samples of e-liquids using gas and liquid chromatography and identified impurities in various brands above the level set for nicotine products in the European Pharmacopoeia [10].

Many countries are still grappling with their approach to regulation for standards of manufacturing, constituents, labelling, marketing, and sales of e-cigarettes [11,12]. In the meantime, a huge range of devices and e-liquids with a wide range of flavors, with few guarantees and limited information for consumers around quality and stability under different conditions, are widely available [8,13]. In 2016, the Food and Drug Administration (FDA) set the requirements for the manufacture of e-liquids in the United States. This includes standards for the accuracy of labelled nicotine content, quality of ingredients in e-liquid, grade-certified bases for these liquids, and minimum standards for the quality of flavors and colors [14,15].

Regulatory guidance from the FDA and International Conference on Harmonization (ICH) requires that drug manufacturers provide forced degradation data to determine the stability and efficacy of a drug under different environmental conditions and to validate drug stability-indicating procedures [16,17,18]. Forced degradation (stress testing) involves subjecting a drug to severe conditions that accelerate the generation of degradation products, enabling the stability of the molecule of interest to be studied [16]. Degradation mechanisms include photolysis, thermolysis, oxidation, and hydrolysis [19].

Several high-performance liquid chromatographic (HPLC) methods are available for the quantification of nicotine [7,20,21,22,23,24,25,26], but only one describes a method for the determination of nicotine in the presence of thermal degradation products [20]. Carlisle et al. studied the effect of thermal degradation on nicotine and nicotine patches using a combination of reversed phase (RP)-HPLC and ion-pair chromatography [20]. There is no literature on the stability of nicotine to other recommended stressors. With an increase in the number and diversity of nicotine products in the market, both regulated and unregulated, this gap needs to be addressed.

In this study, we report a simple, economical, and rapid stability-indicating HPLC method for the quantification of nicotine included within e-liquids.

## 2. Materials and Methods

### 2.1. Materials

We obtained L-nicotine, >99%, pure from Thermo Fisher Scientific New Zealand Limited, Auckland, New Zealand. E-liquids were purchased in six different concentrations and two different flavors from a retail outlet in Auckland, New Zealand. All reagents and solvents used were of analytical reagent (AR) and HPLC grades, respectively. All other chemicals used in the study were at least reagent grade. We obtained the water used in the formulation of buffers by the process of reverse osmosis (0.22 µm Millipore, Burlington, MA, USA).

### 2.2. Chromatographic Conditions and Method Development

We used an Agilent series 1260 HPLC (Agilent Corporation, Waldbronn, Germany), comprising a quaternary pump, vacuum degasser, an autosampler, and a column compartment with thermostat and a photodiode array (PDA) detector, with data acquisition from Chemstation software (Agilent Corporation, Waldbronn, Germany). A Kinetex Evo (core-shell technology) C18 column (150 × 4.6 mm, particle size 5 µm and 100 Å) (Phenomenex, Torrance, CA, USA) was used, with the temperature maintained at 35 °C. After investigating various columns and mobile phases in different combinations, we selected an isocratic method, with the mobile phase consisting of acetonitrile (ACN)-sodium hydrogen carbonate (pH 10.0, 0.03 M) (20:80, *v*/*v*), at a flow rate of 1 mL/min. The UV detection was achieved at 259 nm with a bandwidth of 4 nm. The mobile phases were filtered by using a 0.45 µm filter and degassed by sonication for 10 min before use. The injection volume for the analysis of all samples was 10 µL.

### 2.3. Stress Studies of Nicotine

We determined the stability of pure nicotine and nicotine aqueous solutions under photolytic, thermolytic, hydrolytic (acid/base), and oxidative stress conditions [16]. The forced degradation studies aimed to generate 5% to 20% degradation of nicotine [19]. The purity of the nicotine peaks was tested by using a photo diode array (PDA) detector and Chemstation software by examining ultraviolet (UV) spectra across the nicotine peak. The overlapping of different UV spectra across the peak of interest shows the purity of the peak, and it ensures the absence of degradation peaks within the nicotine peak.

Acid and base hydrolytic stress alongside oxidative stress in the solution form was achieved by dissolving nicotine in aqueous solutions of 0.1 N HCl, 0.1 N NaOH, and H_2_O_2_ (0.003%, 0.3%, and 3% *w*/*v*), respectively, to achieve a concentration of 1 mg/mL. Atmospheric oxygen was not excluded during degradation experiments, and all the solutions were kept in 10 mL glass vials with a small amount of atmospheric headspace. Samples were stored at 60 °C in a Binder Incubator BD240 series (Binder, Tuttlingen, Germany). Initial studies showed oxidative degradation occurred rapidly, so the oxidative degradation was also performed at an ambient temperature of 25 °C. In the pure oily state, nicotine was exposed to ambient light and to a temperature of 60 °C. Samples were withdrawn at predetermined intervals and diluted with mobile phase to obtain a concentration of 20 µg/mL before analysis by HPLC. All the samples, except for photolytic degradation studies, were covered with aluminum foil and stored in the dark. Studies were continued for 10 days or until significant degradation (>5%) was observed.

### 2.4. Method Validation

We validated the developed HPLC method for linearity, range, sensitivity (limits of detection (LOD) and quantitation (LOQ)), accuracy, precision and robustness as per ICH guidelines [17]. To do this, a primary stock solution of nicotine (1 mg/mL) was prepared by dissolving nicotine in mobile phase solvent. The stock solution was serially diluted with mobile phase solvents to produce working solutions in the range of 0.78–50 µg/mL with five replicates. The sensitivity of the HPLC method was determined by calculating LOD and LOQ by injecting serially lower concentrations showing a peak with a signal to noise ratio of at least 3:1 and 10:1, respectively [17]. LOD, the lowest amount of analyte that can be detected by the analytical method, does not need to be quantified [27]. LOQ is the lowest amount of analyte that can be quantified by the analytical method [27]. The accuracy of an analytical method is the closeness between the test results of experimental and actual value. The accuracy of the method is reported as percentage recovery by assay compared to the known added amount. The precision of an analytical method is the closeness between multiple injections of the same sample. It can be expressed as repeatability, intermediate precision, and reproducibility [27]. Intra-day accuracy and precision were calculated by analysis of 3 replicates of 5, 10, 20, and 40 µg/mL. Inter-day accuracy and precision were calculated by analysis of the same concentrations with 3 replicates over 3 days. Instrumental precision or injection repeatability was determined by analyzing 10 injections of one sample of nicotine at a concentration of 20 µg/mL to check the percentage relative standard deviation (% RSD) [18]. The accuracy was calculated by comparing the experimental concentration against the actual concentration [17]. Robustness of an analytical method is its capacity to remain unaffected by small but deliberate variations in the method parameters [17]. In this study, we tested the robustness of our method by varying the mobile phase ratio by ±1%, injection volume from 5 µL to 20 µL, temperature of the column from 30 °C to 40 °C, flow rate from 0.8 mL/min to 1.2 mL/min, and pH of the buffer from 9.5 to 10.5.

### 2.5. Application of the Analytical Method to Analyze E-Liquids and Determine Their Stability at High Temperature

We analyzed e-liquids in a concentration range from 0 mg/mL to 18 mg/mL in two different flavors (mint and watermelon) using the developed HPLC method. E-liquids labelled as 0 mg/mL were analyzed undiluted while other e-liquids were diluted to obtain a concentration of 30 µg/mL before analysis. Additionally, e-liquids were stored in the original containers at a temperature of 60 °C in a Binder Incubator BD240 series (Binder, Germany) and were analyzed after 10 days to determine the stability and ensure the method could differentiate degradation peaks from the analyte peak. The purity of the nicotine peak was calculated using Chemstation software, to ensure the absence of degradation peaks.

## 3. Results

### 3.1. Method Development

**Figure 1:**
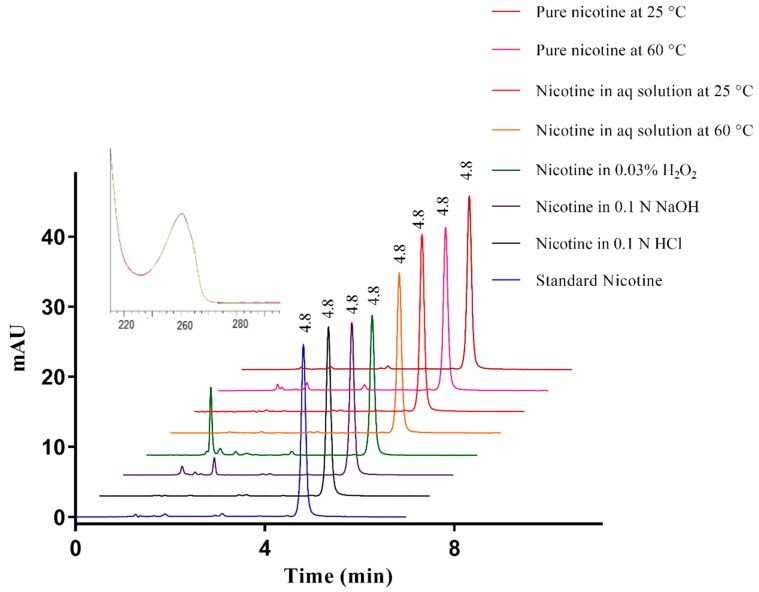
Chromatograms of nicotine standard and in selected stress conditions, starting concentration of 20 µg/mL in all cases. The nicotine peak is seen eluting after 4.8 min in all conditions.

The buffer pH, percentage of the organic phase in the buffer, and column temperature were optimized to achieve chromatographic separation and quantification of nicotine (Figure 1).

**Figure 2:**
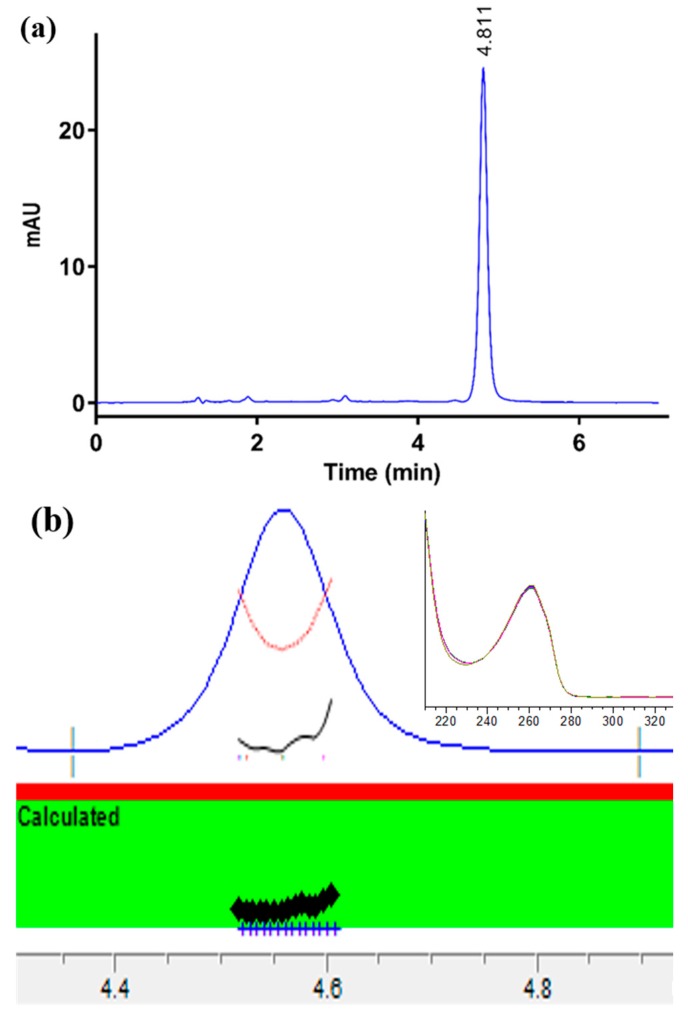
(**a**) Chromatogram of nicotine (20 µg/mL) and (**b**) peak purity profile (red line) of nicotine showing the purity value within the threshold limit. The insert shows the overlapping of different spectra.

The optimum peak separation was achieved using the Kinetex Evo C18 (150 × 4.6 mm, particle size 5 µm and 100 Å) column. After testing various combinations of buffer and organic solvents, a mobile phase consisting of acetonitrile-sodium hydrogen carbonate (NaHCO_3_) (pH 10.0, 30 mM) (20:80, *v*/*v*) with a flow rate of 1 mL/min was used. The column temperature was optimized at 35 °C to achieve the required purity of the nicotine peak. Following a 10 µL injection, the nicotine peak eluted after 4.8 min (Figure 2a) and was detected by a PDA detector at 259 nm. Peak purity was determined by testing between 200 and 400 nm, and the purity index was within the threshold limit (999.499) as demonstrated by a purity ratio (999.741) in the green band and overlapping of different peak spectra, indicating a high degree of similarity (Figure 2b).

### 3.2. Stress Studies of Nicotine

The results of stress studies of nicotine are shown in Table 1.

The degradation experiments were stopped either after 5–20% degradation was observed or after 10 days. Nicotine was stable in the acidic solution at 60 °C, an aqueous medium at 25 °C, and aqueous medium exposed to ambient light. Pure nicotine was stable at 25 °C and when it was exposed to light. However, nicotine degradation was observed in alkaline medium at 60 °C, with 87.7 ± 0.6% of nicotine remaining in the solution after five days. Under oxidative conditions of 3% H_2_O_2_, fast degradation was observed at 60 °C, therefore, the studies were also conducted at lower H_2_O_2_ concentrations and at room temperature. Oxidative stress at elevated temperatures resulted in degradation of nicotine on the first day with 19.6 ± 0.1% and 85.6 ± 0.4% nicotine remaining in 0.3% and 0.03% H_2_O_2_ solutions, respectively. Nicotine degradation was also observed upon oxidative stress at room temperature, with 79.2 ± 0.9% drug remaining after 24 h and 85.7 ± 0.4% drug remaining after 3 days in 0.3% and 0.03% H_2_O_2_, respectively. Aqueous nicotine solution kept at 60 °C showed slow degradation with 83.1 ± 0.2% nicotine remaining on day 10. Pure nicotine also showed some degradation when stored at 60 °C with 93.6 ± 0.2% nicotine remaining after five days. In all cases, the nicotine peak remained pure, indicating nicotine could be separated and quantified in the presence of degradation products.

### 3.3. Method Validation

**Figure 3:**
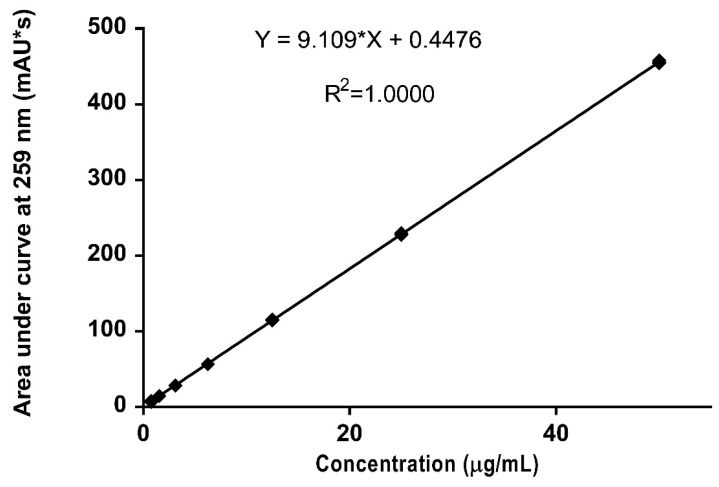
Mean standard HPLC calibration curve of nicotine showing linearity over a concentration range of 0.78–50 µg/mL (*n* = 5, data points indicate mean values and error bars represent standard deviations).

The standard curve of nicotine showed linearity in the range of 0.78–50 µg/mL (Figure 3) with a linear equation of y = 9.1092x + 0.4476 as obtained from the linear regression analysis. The coefficient of correlation (R^2^) was 1.0000.

The LOD and LOQ values were 0.07 µg/mL and 0.3 µg/mL, respectively. The accuracy and precision results have been summarized in Table 2. Intra-day accuracy was between 99.3% and 100.7% with a % RSD of less than 0.9%. Inter-day accuracy was between 100.3% and 100.6% with a % RSD of less than 1.1%.

The developed HPLC method was found to be robust, as evidenced by the accuracy data (Table 3) and a % RSD value less than 0.5%, indicating high precision with variations in the mobile phase, pH of the buffer, flow rate, injection volume, and temperature of the column.

### 3.4. Application of the Analytical Method to Analyze Nicotine Content in E-Liquids and Determine Their Stability at High Temperature

**Figure 4:**
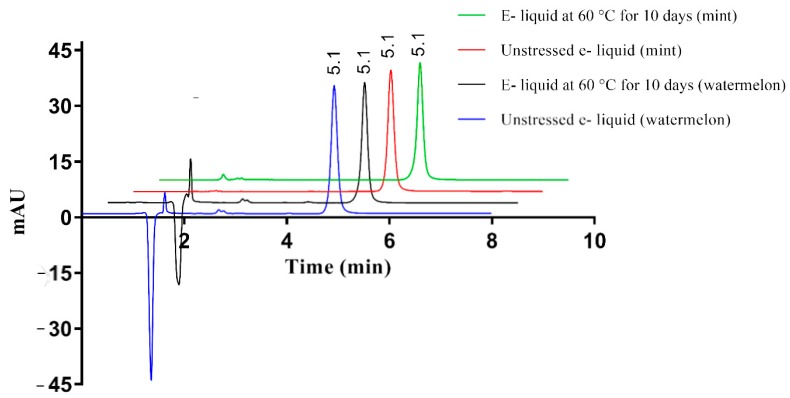
Chromatograms of e-liquids in standard and thermally stressed conditions (30 µg/mL). The nicotine peak is seen eluting after 5 min in all the conditions.−

The HPLC method was able to separate nicotine from the other ingredients present in e-liquids. E-liquids with watermelon flavor showed a huge negative peak followed by another small peak, which could be due to the flavoring agents having lower UV absorbance compared to that of the mobile phase (Figure 4). The peak of nicotine was pure for standard and thermally stressed e-liquid, indicating no degradation products or flavoring agents were hidden under the nicotine peak. Interestingly, the negative peak attributed to the flavoring agent changed after 10 days of storage at 60 °C indicating degradation.

All e-liquids assayed contained nicotine, as advertised, with the exception of the e-liquid labelled 0 mg/mL nicotine, which did not show any nicotine peak. For all other samples, the amount of nicotine present (mg/mL) was higher than the labelled content (Table 4). Variability in the content of nicotine in the e-liquids was observed among different flavors, with the content of nicotine ranging from 112.0% ± 1.52% to 117.9% ± 1.87% that of the labelled values (Table 4). While most e-liquid samples were stable for 10 days at 60 °C, 3 mg/mL nicotine in the mint flavor degraded to 91.3 ± 1.11% remaining.

## 4. Discussion

A stability-indicating HPLC analytical method has been developed and validated to quantify nicotine from a range of samples including e-liquids. The method is rapid, simple in operation, inexpensive, and does not require the use of hazardous chemicals.

We trialed a range of HPLC methods reported in the literature for the quantification of nicotine [7,23,26]. Different columns were trialed, including Gemini (250 × 4.6 mm, 5 µm) (Phenomenex, Torrance, CA, USA) and Kinetex Evo (150 × 4.6 mm, 5 µm) (Phenomenex, CA, USA) columns. Some of the trialed methods showed short retention of 2 to 3 min, and others showed impure peaks. Short retention times of under 4 min for the chemical of interest are undesirable as interference will be experienced with solvent peaks; and the purity of the peak of interest is an important parameter for a stability-indicating HPLC method, to ensure the absence of any degradation peaks within the sample peak. Buffered mobile phase at pH 10.0 was optimized for the analysis of nicotine as most of the nicotine is unionized at this pH (pKa of nicotine = 8.0), which improved its retention on RP-HPLC stationary phase to around 5 min [28].

Chromatograms of nicotine in standard and stress conditions are shown in Figure 1. Nicotine was susceptible to degradation under hydrolytic, oxidative, thermolytic, and photolytic conditions, but the degradation products did not interfere with nicotine analysis. All the validation parameters were within accepted criteria established by regulatory authorities [17]. The proposed method has shown linearity over a concentration range of 0.78–50 µg/mL. The accuracy of the method was found to be within 100 ± 1% and had a high precision with a % RSD < 2. The method was also found to be robust, which showed the reliability of the developed method upon small variations in mobile phase, pH of the buffer, flow rate, column temperature, and injection volume. The developed stability-indicating HPLC method was able to quantify nicotine content from e-liquids, separating nicotine from flavoring agent and thermal degradation products.

Although pure nicotine and aqueous solutions of nicotine showed significant thermal degradation after storage at 60 °C, it is surprising that e-liquids showed only minimal degradation in nicotine content at the same temperature. Pure nicotine liquid showed almost 6.5% reduction in the content of nicotine and an aqueous solution of nicotine showed 17% degradation within 5 and 10 days, respectively, after storage at 60 °C. Among the e-cigarette solutions analyzed, only one sample of 3 mg/mL (mint flavored) showed more than 5% degradation. While some degradation was observed in the other samples, this was less than 5%. One possible explanation is the presence of other ingredients exerting a stabilizing effect on the formulations. According to the stability studies, thermal degradation occurs in aqueous solutions due to hydrolysis. The polypropylene and glycerol used in the e-liquids are known to be hygroscopic, and by introducing water to the system the nicotine would be more prone to degradation. If the formulation had acquired water from the atmosphere or during preparation, this would explain why 3 mg/mL (mint flavored) degraded faster than the other samples. A small peak at around 1.5 min appeared for thermally stressed mint flavored e-liquid, indicating the formation of degradation products. This potential highlights the importance of manufacturing practices in the preparation of e-liquids. In addition to nicotine degradation, the reduction in the area of negative peak in thermally-stressed watermelon samples indicates the flavoring agents is degrading. Concerningly, the safety of degradation products of nicotine or other ingredients is not known.

## 5. Conclusions

We developed and validated a stability-indicating HPLC method for the quantification of nicotine. The simple and rapid method can be used for the routine analysis of nicotine and is suitable to be adopted in different laboratories. It is of concern that in all e-liquids tested, the actual amount of nicotine was higher than the labelled amount. The stability-indicating method has good potential to be used as an analytical tool to determine the actual content and stability of various regulated and unregulated nicotine products available in the market.

## Figures and Tables

**Table 1:** Stress studies of nicotine showing % nicotine remaining after exposed to various stressed conditions.

Stress Conditions	Duration	(%) Drug Remaining
Acidic degradation (0.1 N HCl, 60 °C)	10 days	97.0 ± 0.9
Alkaline degradation (0.1N NaOH, 60 °C)	5 days	87.8 ± 0.6
Oxidative degradation (3% H_2_O_2_, 60 °C)	1 h	0
Oxidative degradation (0.3% H_2_O_2_, 60 °C)	1 h	19.6 ± 0.1
Oxidative degradation (0.3% H_2_O_2_, 25 °C)	1 h	79.2 ± 0.9
Oxidative degradation (0.03% H_2_O_2_, 60 °C)	1 h	85.6 ± 0.4
Oxidative degradation (0.03% H_2_O_2_, 25 °C)	3 days	86.7 ± 0.4
Aqueous solution (60 °C)	10 days	83.1 ± 0.2
Aqueous solution (25 °C)	10 days	100.9 ± 0.1
Aqueous solution (25 °C, ambient light)	10 days	99.7 ± 0.5
Pure nicotine (60 °C)	5 days	93.6 ± 0.2
Pure nicotine (25 °C)	10 days	96.2 ± 0.4
Pure nicotine (25 °C, ambient light)	10 days	95.2 ± 0.3

**Table 2:** Accuracy and precision data of the HPLC method obtained from intra- and inter-day studies. Concentrations shown in µg/mL. RSD = relative standard deviation.

Conc. (µg/mL)	Intra-Day		Inter-Day	
	Accuracy (%)	Precision (% RSD)	Accuracy (%)	Precision (% RSD)
5	100.7	0.70	100.5	1.08
10	99.4	0.89	100.5	0.93
20	99.3	0.88	100.6	0.97
40	100.0	0.61	100.3	0.38

**Table 3:** Robustness determined by the influence of changes in chromatographic parameters on the reversed phase (RP)-HPLC method of nicotine.

Parameters	Conditions	Accuracy (%)	Precision (% RSD)
Mobile Phase	Buffer 79% ACN 21%	100.1	0.49
	Buffer 80% ACN 20%	98.5	0.21
	Buffer 81% ACN 19%	99.4	0.48
pH of buffer	9.5	99.5	0.46
	10.0	98.5	0.49
	10.5	100.0	0.28
Flow rate	0.8 mL/min	103.3	0.28
	1.0 mL/min	102.6	0.19
	1.2 mL/min	102.4	0.35
Column temperature	30 °C	102.4	0.35
	35 °C	102.6	0.19
	40 °C	103.1	0.28
Injection volume	10 µL	102.6	0.19
	20 µL	102.9	0.10
	30 µL	103.5	0.11

**Table 4:** Nicotine concentration analysis for standard and thermally stressed e-liquids.

Flavour	Labelled Nicotine Concentration (mg/mL)	Actual Content Expressed as a % of the Labelled Content of Nicotine in E-Liquid (±SD)	% Nicotine Remaining in Samples after 10 Days Storage at 60 °C (±SD)
Mint	0	0.0 ± 0.00	0 ± 0.00
Mint	3	112.0 ± 1.52	91.3 ± 1.11
Mint	6	116.7 ± 0.47	95.7 ± 1.21
Mint	9	112.1 ± 0.57	97.7 ± 0.50
Mint	12	115.3 ± 2.63	94.0 ± 4.20
Mint	18	115.9 ± 0.58	98.2 ± 0.50
Watermelon	0	0.0 ± 0.00	0.0 ± 0.00
Watermelon	3	117.9 ± 1.87	96.9 ± 0.30
Watermelon	6	115.4 ± 0.63	98.9 ± 0.68
Watermelon	9	116.2 ± 0.39	98.3 ± 0.23
Watermelon	12	115.4 ± 1.51	97.7 ± 0.38
Watermelon	18	115.2 ± 0.74	99.4 ± 1.40
